# Osteopenia Metabolomic Biomarkers for Early Warning of Osteoporosis

**DOI:** 10.3390/metabo15010066

**Published:** 2025-01-20

**Authors:** Jie Wang, Dandan Yan, Suna Wang, Aihua Zhao, Xuhong Hou, Xiaojiao Zheng, Jingyi Guo, Li Shen, Yuqian Bao, Wei Jia, Xiangtian Yu, Cheng Hu, Zhenlin Zhang

**Affiliations:** 1 Department of Osteoporosis, Metabolic Bone Disease and Genetic Research Unit, Shanghai Sixth People’s Hospital Affiliated to Shanghai Jiao Tong University School of Medicine, Shanghai 200233, China; 2 Department of Endocrinology and Metabolism, Shanghai Sixth People’s Hospital Affiliated to Shanghai Jiao Tong University School of Medicine, Shanghai Diabetes Institute, Shanghai Clinical Center of Diabetes, Shanghai Key Laboratory of Diabetes Mellitus, Shanghai Key Clinical Center for Metabolic Disease, Shanghai 200233, China; 3 Clinical Research Center, Shanghai Sixth People’s Hospital Affiliated to Shanghai Jiao Tong University School of Medicine, Shanghai 200233, China; 4 Center for Translational Medicine, and Shanghai Key Laboratory of Diabetes Mellitus, Department of Endocrinology and Metabolism, Shanghai Sixth People’s Hospital Affiliated to Shanghai Jiao Tong University School of Medicine, Shanghai 200233, China; 5 Shanghai Diabetes Institute, Shanghai Key Laboratory of Diabetes Mellitus, Shanghai Clinical Center for Diabetes, Shanghai Sixth People’s Hospital Affiliated to Shanghai Jiao Tong University School of Medicine, Shanghai 200233, China; 6 Hong Kong Phenome Research Centre, School of Chinese Medicine, Hong Kong Baptist University, Hong Kong 999077, China

**Keywords:** bone mineral density, osteopenia, metabolomics, biomarkers, early warning

## Abstract

**Introduction**: This study aimed to capture the early metabolic changes before osteoporosis occurs and identify metabolomic biomarkers at the osteopenia stage for the early prevention of osteoporosis. **Materials and Methods**: Metabolomic data were generated from normal, osteopenia, and osteoporosis groups with 320 participants recruited from the Nicheng community in Shanghai. We conducted individual edge network analysis (iENA) combined with a random forest to detect metabolomic biomarkers for the early warning of osteoporosis. Weighted Gene Co-Expression Network Analysis (WGCNA) and mediation analysis were used to explore the clinical impacts of metabolomic biomarkers. **Results**: Visual separations of the metabolic profiles were observed between three bone mineral density (BMD) groups in both genders. According to the iENA approach, several metabolites had significant abundance and association changes in osteopenia participants, confirming that osteopenia is a critical stage in the development of osteoporosis. Metabolites were further selected to identify osteopenia (nine metabolites in females; eight metabolites in males), and their ability to discriminate osteopenia was improved significantly compared to traditional bone turnover markers (BTMs) (female AUC = 0.717, 95% CI 0.547–0.882, versus BTMs: *p* = 0.036; male AUC = 0.801, 95% CI 0.636–0.966, versus BTMs: *p* = 0.007). The roles of the identified key metabolites were involved in the association between total fat-free mass (TFFM) and osteopenia in females. **Conclusion**: Osteopenia was identified as a tipping point during the development of osteoporosis with metabolomic characteristics. A few metabolites were identified as candidate early-warning biomarkers by machine learning analysis, which could indicate bone loss and provide new prevention guidance for osteoporosis.

## 1. Introduction

Osteoporosis is a systemic osteopathy characterized by decreased bone mass and strength, which leads to increased bone fragility and fracture. As one of the most common metabolic bone diseases, osteoporosis currently affects 10.9 million men and 49.3 million women in China [1]. Besides clinical symptoms, a serious consequence of osteoporosis is osteoporotic fractures, which have a high morbidity and mortality [2,3]. As a chronic disorder of bone metabolism, osteoporosis is preventable and treatable; however, osteoporosis is always silent in its early stages, such that only a small proportion of patients can be evaluated and diagnosed in a timely manner. Osteoporosis is a dynamic process; lifestyle interventions and pharmacotherapies, such as calcium, vitamin D, and bisphosphonates, are all beneficial for people with osteopenia and osteoporosis and some people at high risk of osteoporosis [4,5]. According to previous research, patients with osteoporosis have a higher fracture risk, but the fracture rate in adults with osteopenia is higher due to the larger osteopenia patient base [6]. Randomized controlled trials have demonstrated that treatment for individuals with osteopenia can increase bone density and prevent the occurrence of fractures [7,8]. Therefore, osteopenia, as the precursor stage of osteoporosis, is a vital stage for preventing osteoporosis and fractures.

Osteoporosis is generally accompanied by a decrease in bone mineral density (BMD). Low BMD is a strong risk factor for fractures, and the decrease in BMD is associated with the increase in the risk of fractures [9,10]. Dual-energy X-ray absorptiometry (DXA) scanning, which is the gold standard for BMD measurement and fracture risk assessment (FRAX), forms the basis of diagnosis, risk prediction, and curative effect evaluation for osteoporosis. BMD is a good predictor of future fracture risk, but it can only reflect the current bone density status, not its quality, which is limited to the comprehensive assessment of the patients. About half of patients with osteopenia or even a normal BMD suffer fragility fractures [11,12]. FRAX is based on relevant clinical risk factors such as sex, age, body weight, height, fracture history, and lifestyle with or without BMD. It is convenient for identifying patients with a high risk for osteoporotic fracture, but its sensitivity and discriminative capacity are suboptimal [13,14]. Since bone health is an integration of both bone mass and bone quality, we need more comprehensive assessment tools or novel biomarkers to evaluate bone inner quality, which may be independent of or combined with BMD and FRAX. The identification of such novel biomarkers is urgently needed for the early diagnosis of osteoporosis and prevention of osteoporotic fractures.

Bone health largely depends on the metabolic state of the human body. The human skeleton is a multifunctional organ that undergoes continuous remodeling, including bone resorption by osteoclasts and bone formation by osteoblasts. Generally, there is an exquisite balance between bone resorption and bone formation, and bone loss occurs when this balance is broken following a series of bone metabolic disorders. During bone remodeling, there are some protein or protein derivative metabolites released by osteoblasts or osteoclasts, which can reflect the dynamic bone remodeling process; these are referred to as bone turnover markers (BTMs). BTMs reflect the status of bone metabolism since they respond rapidly to changes in bone physiology. Based on present research, BTMs are highly correlated with BMD and are promising tools for the prediction of osteoporotic fracture risk [15,16]. BTMs are also considered useful for the assessment of patient compliance and for assessing the efficacy of therapies [17,18]. Along with the development of the human phenome project, the phenome-based actionable P4 medicine strategy is attractive in proactive intervention [19]. In particular, metabolomics, by allowing the simultaneous assessment of multiple metabolites in biological fluids [20], may be suited to studying the dynamic process of bone remodeling. Such metabolite profiling strategies have been implemented to identify metabolic biomarkers in people with osteoporosis [21]. Currently, there are some studies which indicate that metabolic abnormalities are closely associated with osteoporosis and osteopenia [22,23]. These findings can add new insights into the understanding of the metabolomic alterations of osteoporosis. In addition, identifying biomarkers specifically for osteopenia will be meaningful in the candidate biomarker discovery for predicting osteoporosis progression. However, there are inconsistencies in these results due to the small sample sizes and differences in research designs and metabolomic detection methods.

Our study evaluated the association between serum metabolites and BMD, aiming to identify early metabolic biomarkers before osteoporosis occurs that, as such, may be indicative of osteoporosis risk and be beneficial for the prevention of osteoporosis. Furthermore, we assessed the predictive ability of the metabolites identified and examined their correlation with conventional osteoporosis clinical factors, in order to provide insights into the underlying pathogenesis of bone metabolism.

## 2. Materials and Methods

### 2.1. Participants and Data Collection 

#### 2.1.1. Participants

Our participants were all from the Nicheng community in the Shanghai area. Participants with diseases or medications that might influence bone metabolism were excluded. Gender, age, and body mass index (BMI) were then matched in the included participants. Finally, a total of 320 participants (138 males and 182 postmenopausal females) were recruited. The basic information of these participants was previously described [24]. All participants provided written informed consent. Our study was approved by the Institutional Review Board of the Shanghai Jiao Tong University Affiliated Sixth People’s Hospital with the approval number 2015-KY-002(T) and 2015-27-(1).

#### 2.1.2. Clinical Measurements

Anthropometric indices (age, height (m), and weight (kg)) were recorded. BMI was calculated as weight/height2. Biochemical indices, including plasma glucose, liver function, renal function, fasting serum total cholesterol (TC), low-density lipoprotein cholesterol (LDL), high-density lipoprotein cholesterol (HDL), and triglyceride, apolipoproteins, and albumin levels, were collected in the fasting status and assessed with a type 7600-020 Automated Analyzer (Hitachi, Tokyo, Japan). HbA1c values were measured with a Bio-Rad Variant II hemoglobin testing system (Bio-Rad Laboratories, Hercules, CA, USA). Thyroid-stimulating hormone (TSH), free triiodothyronine (FT3), and free thyroxine (FT4) were all detected by the chemiluminescence method (Roche cobas e801). Using an automated Roche electrochemiluminescence system (Roche Diagnostics GmbH, Mannheim, Germany), the BTMs (osteocalcin, PINP, and β-CTX), serum 25-hydroxyvitamin D3 [25(OH)D3], and parathyroid hormone (PTH) were measured. Serum insulin was assessed with an automatic electrochemiluminescence analyzer (Cobase 601) (Roche Company, Wyhlen, Germany). Insulin sensitivity indices such as HOMA-IR, HOMA-IS and GUTT-ISI, beta cell function indices, including HOMA-β, the Stumvoll first-phase insulin secretion index (STU-1), and the Stumvoll second-phase insulin secretion index (STU-2) were calculated based on the above clinical indicators [25,26]. Body composition, including body fat percentage (BFP), total fat mass (TFM), and total fat-free mass (TFFM), was assessed using the BC-420 Tanita Body Composition Analyzer (Tanita, Tokyo, Japan). Abdominal visceral fat area (VFA) and subcutaneous fat area (SFA) were acquired at the abdominal level between the fourth and fifth lumbar vertebrae with subjects in the supine position using a 3.0-T magnetic resonance imaging (MRI) system (Achieva; Philips, Best, The Netherlands) and calculated using image analysis software (SLICEOMATIC, version 4.2; Tomovision, Inc., Montreal, QC, Canada). The BMDs of the lumbar spine (L1-4), femoral neck, and total hip were determined by DXA (Hologic QDR-2000, Hologic Corporation, Waltham, MA, USA). The T-score was calculated using the following formula: (measured BMD-Young adult mean BMD)/Young adult population standard deviation (SD) [27]. Participants with T-scores less than −2.5 SD at any site were included in the osteoporosis group, participants with T-scores greater than −2.5 SD at all sites were included as controls, and other participants were included in the osteopenia group [28].

#### 2.1.3. Sample Collection and Metabolomic Profiling

Fasting blood samples were collected in tubes containing sodium ethylenediaminetetraacetic acid (EDTA). Serum samples were collected after centrifugation at room temperature for 1 h and centrifuged at 3000 rpm for 10 min, and then they were stored at −80 °C. Before being assayed, the samples were thawed and centrifuged at 4 °C at 12,000 rpm for 10 min. All measurements were performed with an AbsoluteIDQ ™ p180 Kit (BIOCRATES Life Sciences AG, Innsbruck, Austria). The entire assay workflow included sample registration, automated calculation of metabolite concentrations (FIA-MS/MS), import of LC-MS/MS data from Analyst, and export of data into other analysis programs, all driven by the Biocrates proprietary Met*IQ* ^TM^ software (1.2.Ob-DB92-Lithium-1563) of the kit. Using the AbsoluteIDQ ^TM^ p180 Kit, a total of 221 metabolites from five groups (acylcarnitines, amino acids, biogenic amines, lipids, and hexose) were quantified. Some lipid standards were not commercially available, so we used semi-quantitation based on isotopic lipid internal standards for lipid quantitation.

### 2.2. Differential Metabolite Analysis (DMA)

**Figure 1:**
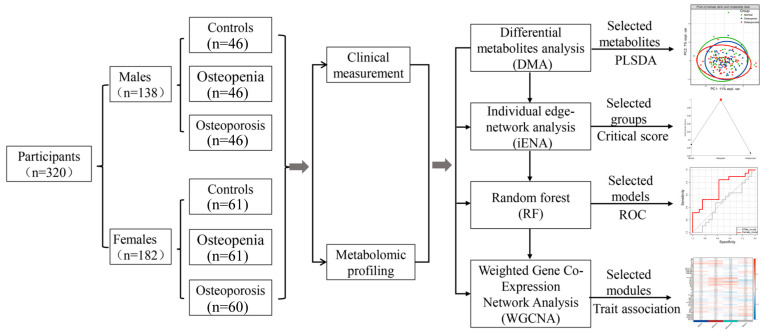
Analysis procedure used in this study. The osteopenia state is critical in distinguishing patients with pre-osteoporosis.

The detailed analysis procedure is summarized in Figure 1. The participants were divided into three groups: a normal control/healthy group, an osteoporosis group, and an osteopenia group. Metabolite data missing more than 20% of the data were excluded. We assessed the similarities in the different sample groups based on metabolite levels by a principal component analysis (PCA) and a partial least squares discriminant analysis (PLSDA) for all metabolites (using the R 4.0.0 software package mixOmics). We carried out a test for trends among the three ordered groups, namely controls->osteopenia->osteoporosis group, using the Jonckheere–Terpstra test. Differential metabolite analysis between any two groups was conducted using Wilcoxon’s rank sum test. The level of significance was set at *p* < 0.05.

### 2.3. Individual Edge Network Analysis (iENA)

We previously proposed an individual-based computational framework (i.e., iENA) as a powerful network analysis tool to quantify disease progression [29]. Recently, we also used an adjusted iENA method using samples from healthy individuals as a network reference in a proof-of-concept study on microbiota dynamics [30,31]. Here, we used an improved approach to analyze the key metabolic biomarkers and networks to quantify different disease states using metabolomic data, combined with other network analysis methods to correlate metabolic biomarkers with conventional osteoporosis clinical factors.

We constructed a metabolic edge network using iENA in three steps. First, using the mean value of reference samples, a co-expression network of metabolites for one sample was built with a single-sample measurement of the Pearson correlation coefficient (sPCC) [29]. Then, the top-ranked sPCC edges (i.e., one pair of metabolites) were selected as the background “nodes” for constructing the subsequent edge network because of the absence of a background (association or interactive) network for metabolites. Finally, the fourth-order correlation coefficient for each edge pair (i.e., two metabolite pairs) was quantified by shPCC [29] for each single sample.

According to the theory of dynamic network biomarkers [32], we only focused on the correlations between the preselected high-ranked relations (edges) to obtain the closely related functional metabolite groups corresponding to each sample from the different disease states. Furthermore, we identified and quantified individual-specific metabolic biomarkers. It would be expected that each of these individual-specific biomarkers should be related to the clinical phenotype to some extent because the closely contacted candidate metabolites are identified in a certain disease state, e.g., a critical state like osteopenia. The top-ranked edge pairs are edge biomarkers that have strong high-order abundance correlations and can be viewed as feature candidates representing a set referred to as “domain” metabolites. We extended the warning signal (i.e., CI index) of the dynamic network biomarker model [32,33,34], using a single-sample measurement [that is, a composite index (sCI)] to quantify the disease state of each sample. This was used to indicate disease early-warning signals when the values were sufficiently large.

$$sCI=\frac{\bar{{sPCC}_{in}}}{\bar{{sPCC}_{out}}}\times \bar{s{SD}_{in}}\;sCI=\frac{\bar{\sum_{x,y\in domain}\left|sPCC\left(x,y\right)\right|}}{\bar{\sum_{x\in domain,y\notin domain}\left|sPCC\left(x,y\right)\right|}}\times \bar{\sum_{x\in domain}\left|x-u_{x}\right|}$$

where *sPCC_in_* is the average absolute value of *PCC* in the domain metabolic group in one sample; *sPCC_out_* is the average absolute value of *PCC* between the domain metabolic group and the other metabolites in one sample; and *sSD_in_* is the average standard deviation in the domain metabolic group.

### 2.4. Random Forest (RF)

As a supervised classifier, random forest (RF) works in an ensemble manner. It consists of many decision trees and establishes a joint classification model to summarize the votes from its member trees to determine the output categories/classes of new samples. In RF, every decision tree is grown on one bootstrap sample set with a randomly selected feature subset. The construction of the RF involves training all of its member decision trees. Considering the existence of certain differences between different decision trees, RF usually averages the predictions from prior-trained decision trees on a new sample to obtain the final prediction. In fact, RF is a well-known machine learning approach and has been widely adopted in biological and biomedical studies [35,36]. The R package randomForest was used to build the RF for metabolite profiles in this work. Similar to our previous workflow [24], based on the differential metabolites from the above DMA, the datasets from the normal control and osteopenia groups were divided into training data and testing data, where 70% of the samples were training data and the remaining 30% were testing data. First, the RF learned using the training data and identified key metabolites as biomarkers based on their importance scores in the RF. Then, the RF learned again using the training data with the metabolite biomarkers identified. Finally, the RF was validated on the test data using area under the curve (AUC) measurements from receiver operating characteristic (ROC) curves.

### 2.5. Weighted Gene Co-Expression Network Analysis (WGCNA)

WGCNA is a widely used omics data analysis approach for co-expression networks and modules [37]. For the metabolite data analyzed here, WGCNA can help infer the co-expressed associations among different metabolites and extract groups of closely interactive metabolite modules. Furthermore, using the trait association test supplied in WGCNA, it can also calculate the correlation between metabolite modules and clinical indices and can select metabolite modules with significant clinical meaning, which helps explain the clinical relevance and importance of certain metabolite modules and indicate the key roles of a few metabolite biomarkers in these modules.

### 2.6. Mediation Analysis

To explore the role of the identified key metabolites in osteoporosis progression, we conducted mediation analysis to evaluate whether the metabolites (mediator) mediated the relationships between clinical characteristics (exposure) and osteopenia or osteoporosis (outcome). We comprehensively assessed all clinical characteristics as exposures and the metabolites identified in the RF model as mediators in the mediation analysis. Factors with significant total and indirect effects were defined as having mediating effects. The mediating role of each metabolite was examined separately by estimating the indirect effect and direct effect of the associations through two logistic models (1. exposure → outcome; 2. exposure + mediator → outcome) using the mediation R package.

## 3. Results

### 3.1. Characterization of Osteopenia as a Unique Stage Before Osteoporosis

**Figure 2:**
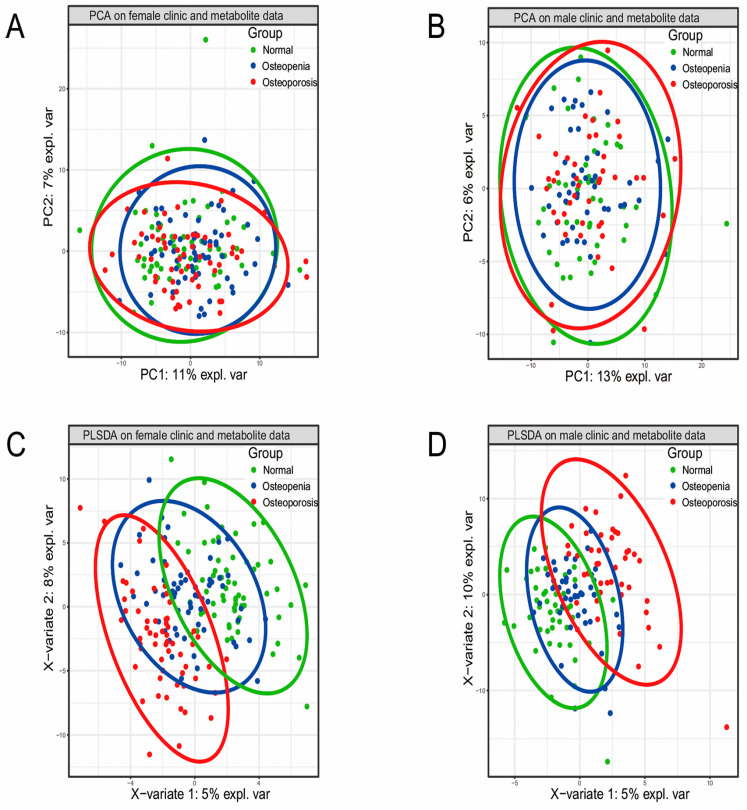

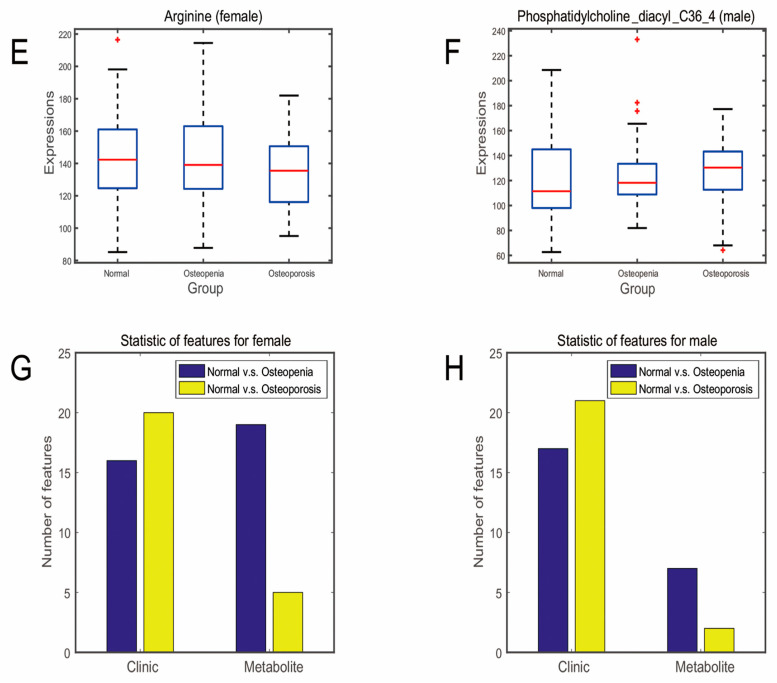
Differential analysis of metabolite profiles. (**A**) PCA of the data from females. (**B**) PCA of the data from males. (**C**) PLSDA analysis of the data from females. (**D**) PLSDA analysis of the data from males. (**E**) A representative metabolite showing a tendency to change levels in the different female groups. (**F**) A representative metabolite showing a tendency to change levels in the different male groups. (**G**) Statistics of the differential clinical symptoms and differential metabolites for the different female groups. (**H**) Statistics of the differential clinical symptoms and differential metabolites for the different male groups.

PCA and PLSDA based on all metabolites were performed in the three BMD groups. According to PCA (Figure 2A,B) and PLSDA (Figure 2C,D), the metabolic profiles of the osteopenia group overlapped with the other two groups. In contrast, the division between the control group and the osteoporosis group was relatively clear. The results of the trend test showed that some metabolites increased or decreased going from one stage to another, which may reflect disease development (Figure 2E,F). A total of 13 metabolites in females and 8 metabolites in males were found in the trend test and are listed in Table S1. We further carried out a differential metabolite analysis between any two groups and found that there were very distinct metabolite differences between the control group and the osteopenia group, although the clinical symptoms between the control group and osteoporosis group were more obvious (Figure 2G,H). These results suggest that osteopenia might be a critical state/stage during the osteoporosis development process.

### 3.2. Detection of Osteopenia as a Critical Warning Signal Before Osteoporosis by iENA

**Figure 3:**
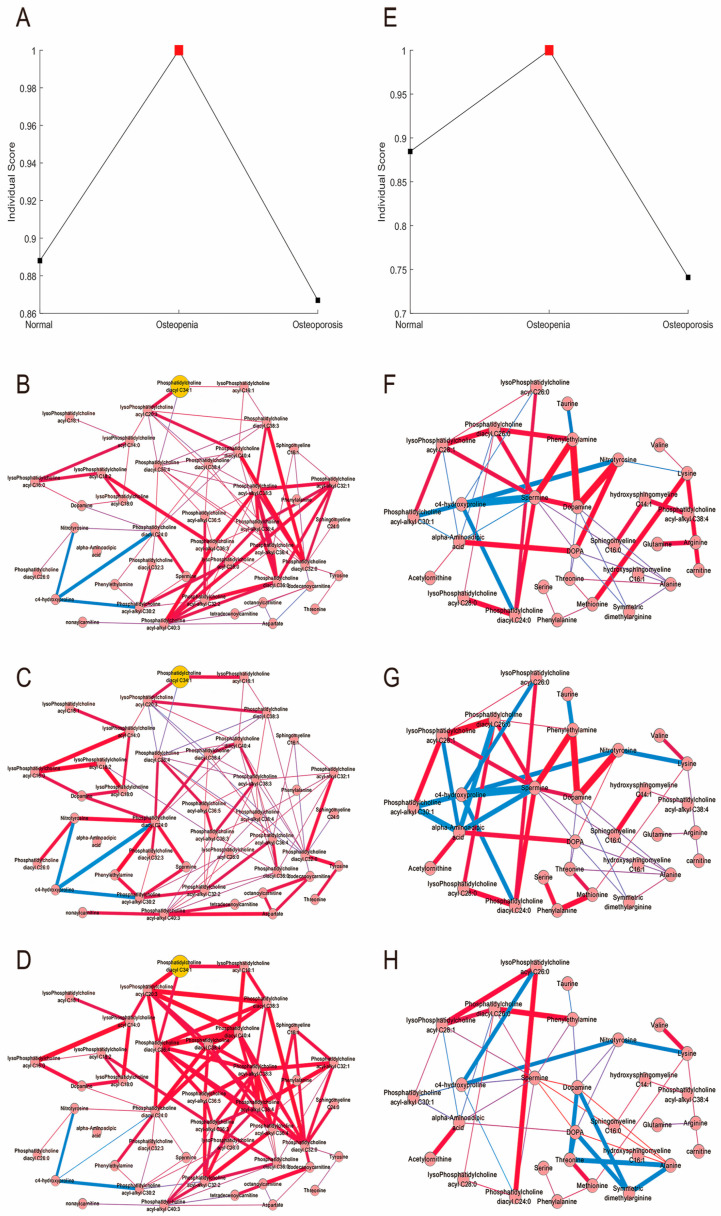
Individual edge network analysis. (**A**) The early-warning index change for the three female groups. (**B**) The co-expressed metabolite biomarker network for the female control group. (**C**) The co-expressed metabolite biomarker network for the female osteopenia group. (**D**) The co-expressed metabolite biomarker network for the female osteoporosis group. (**E**) The early-warning index change for the three male groups. (**F**) The co-expressed metabolite biomarker network for the male control group. (**G**) The co-expressed metabolite biomarker network for the male osteopenia group. (**H**) The co-expressed metabolite biomarker network for the male osteoporosis group.

To test the critical state during biological processes in bone metabolism, we performed iENA at the different disease developmental stages. The composition index (sCI) value, serving as a quantitative measurement for assessing the critical state, reached a maximum in both female and male samples in the osteopenia group, which would participate in the early dysfunction of the metabolite network and cause the subsequent bone disease. We also selected the metabolite module with the highest sCI as the “domain” metabolites, which were identified through a computational framework including metabolite filtering; metabolite co-expression network reconstruction; metabolite module recognition; and critical metabolite module evaluation (Figure 3A,E). Here, alterations in metabolites were more obvious in the osteopenia group, suggesting that there are intense and variable metabolic changes at this stage. Then, we reconstructed the co-expressed metabolite networks in different BMD groups separately for females and males, thus finding several unique metabolic changes in the osteopenia stage for both genders. As shown in Figure 3B–D, the connections between phosphatidylcholine diacyl C24:0 and phosphatidylcholine diacyl C32:3, phenylethylamine and phosphatidylcholine acyl-alkyl C30:2, and tyrosine and threonine became more significant in the osteopenia stage. As shown in Figure 3F–H, the correlations of phosphatidylcholine diacyl C26:0 with alpha-Aminoadipic acid and lysoPhosphatidylcholine acyl C28:0 and those of alpha-Aminoadipic acid with lysoPhosphatidylcholine acyl C28:1, Phosphatidylcholine acyl-alkyl C30:1, and Spermine all became more significant in the osteopenia stage. These unique correlations in the osteopenia stage are mainly involved in phosphatidylcholine and amino acid metabolism. At this stage, a group of key metabolites, i.e., “domain” metabolites, is predicted to drive the development of osteoporosis. The 35 key metabolites in males and 48 key metabolites in females are listed in Table S2.

### 3.3. Identification of Metabolomic Biomarkers of Pre-Osteoporosis for Early Warning of Osteoporosis by RF

After identifying the important role of osteopenia, it then became necessary to identify metabolite biomarkers that could be used to provide a new predictive model for the early diagnosis of osteopenia that could then be used for the early prevention of osteoporosis. With regard to differential metabolites in DMA between the control group and osteopenia group, 13 metabolites in males and 31 metabolites in females (shown in Table S3) were identified. Then, these metabolites were further tested using the RF algorithm to classify the normal control and osteopenia groups.

**Figure 4:**
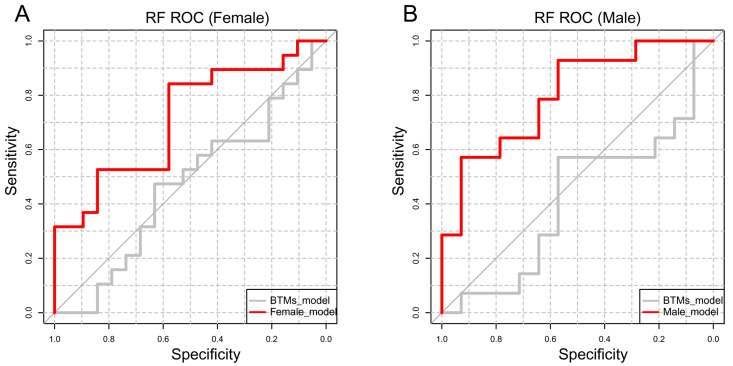
ROC curves of RF models for the diagnosis of osteopenia. (**A**) Metabolite model and BTM model for females. BTM model: AUC = 0.468, 95% CI 0.278–0.658; female model: AUC = 0.717, 95% CI 0.547–0.882; female model versus BTMs: *p* = 0.036. ROC: receiver operating characteristic; BTMs: bone turnover markers; AUC: area under the curve; CI: confidence interval. BTMs include osteocalcin, N-terminalprocollagen of type I collagen (PINP), and β-cross-linked C-telopeptide of type I collagen (β-CTX). The female model includes phosphatidylcholine diacyl C34:1, phosphatidylcholine diacyl C38:5, phosphatidylcholine diacyl C40:5, phosphatidylcholine diacyl C40:6, phosphatidylcholine acyl alkyl C36:1, phosphatidylcholine acyl alkyl C36:4, phosphatidylcholine acyl alkyl C38:5, phosphatidylcholine acyl alkyl C40:5, and phosphatidylcholine acyl alkyl C42:2. (**B**) Metabolite model and BTM model for males. BTM model: AUC = 0.418, 95% CI 0.195–0.642; male model: AUC = 0.801, 95% CI 0.636–0.966; male model versus BTMs: *p* = 0.007. ROC: receiver operating characteristic; BTMs: bone turnover markers; AUC: area under the curve; CI: confidence interval. BTMs include osteocalcin, N-terminal procollagen of type I collagen (PINP), and β-cross-linked C-telopeptide of type I collagen (β-CTX). The male model includes lyso-phosphatidylcholine acyl C16:0, phosphatidylcholine diacyl C34:1, sphingomyelin C26:1, arginine, glutamine, ornithine, valine, and spermine.

Based on RF, our ability to predict osteopenia was further improved with these key metabolites. The area under the curve (AUC) of the receiver operating characteristic (ROC) curves increased significantly compared to the traditional BTM model (BTM: AUC = 0.468, 95% CI 0.278–0.658; female model: AUC = 0.717, 95% CI 0.547–0.882; female model versus BTMs: *p* = 0.036) in females (Figure 4A). Similar results (Figure 4B) were also observed in males (BTM: AUC = 0.418, 95% CI 0.195–0.642; male model: AUC = 0.801, 95% CI 0.636–0.966; male model versus BTMs: *p* = 0.007). Metabolites with the highest importance (≥5%) were selected as potential early-warning predictors (eight metabolites in males, including lysophosphatidylcholine acyl C16:0, phosphatidylcholine diacyl C34:1, sphingomyelin C26:1, arginine, glutamine, ornithine, valine, and spermine; and nine metabolites in postmenopausal females, including phosphatidylcholine diacyl C34:1, phosphatidylcholine diacyl C38:5, phosphatidylcholine diacyl C40:5, phosphatidylcholine diacyl C40:6, phosphatidylcholine diacyl C36:1, phosphatidylcholine acyl alkyl C36:4, phosphatidylcholine acyl alkyl C38:5, phosphatidylcholine acyl alkyl C40:5, and phosphatidylcholine acyl alkyl C42:2).

### 3.4. Investigation of Metabolite–Clinical Associations at the Metabolomic Module and Biomarker Levels

**Figure 5:**
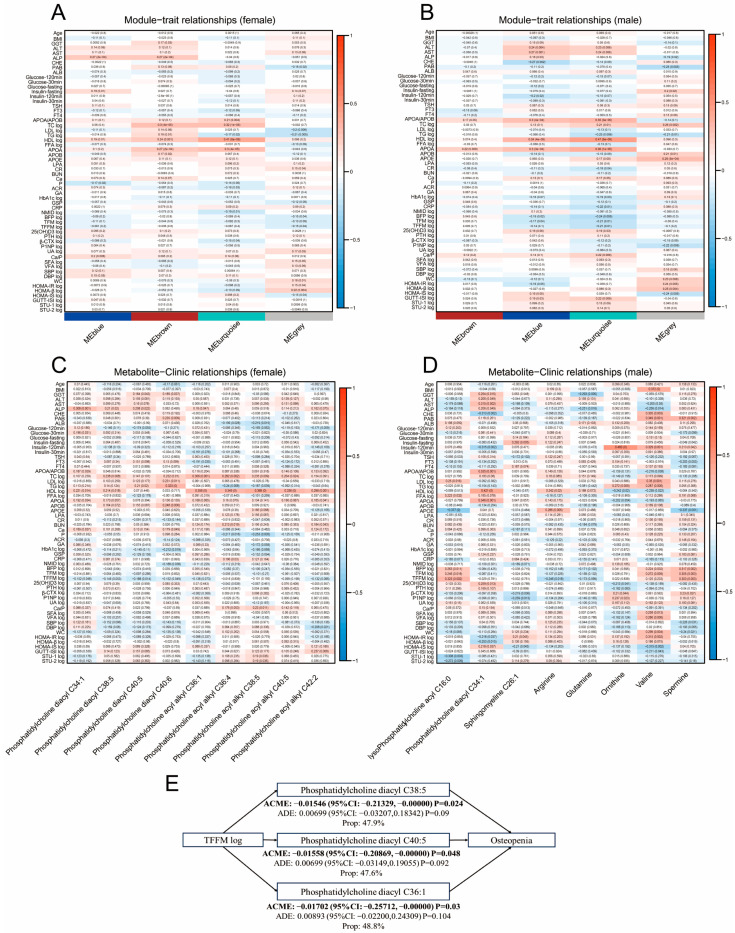
Association between metabolites and clinical indices. –––(**A**) Association between metabolomic modules and clinical indices (females). (**B**) Association between metabolomic modules and clinical indices (males). (**C**) Association between metabolomic biomarkers and clinical indices (females). (**D**) Association between metabolomic biomarkers and clinical indices (males). (**E**) Mediation analysis of metabolites between TFFM and osteopenia in females. BMI, body mass index; GGT, γ-glutamyl transpeptidase; ALT, alanine aminotransferase; AST, aspartate aminotransferase; ALP, alkaline phosphatase; CHE, cholinesterase; PAB, prealbumin; ALB, albumin; TSH, thyroid stimulating hormone; FT3, free tri-Iodothyronine; FT4, free thyroxine; APO, apolipoprotein; TC, total cholesterol; LDL, low-density lipoprotein cholesterol; TG, triglyceride; HDL, high-density lipoprotein cholesterol; FFA, free fatty acid; LPA, Lipoprotein(a); CR, creatinine; BUN, blood urea nitrogen; Ca, calcium; P, phosphorus; ACR, albumin-to-creatinine ratio; GA, glycated albumin; HbA1c, glycated hemoglobin; GSP, glycosylated serum protein; CRP, C-reactive protein; NMID, N-terminal osteocalcin; BFP, body fat percentage; TFM, total fat mass; TFFM, total fat-free mass; 25(OH)D, 25-hydroxyvitamin D3; PTH, parathyroid hormone; β-CTX, β-cross-linked C-telopeptide of type I collagen; PINP, N-terminalprocollagen of type I collagen; UA, uric acid; SFA, subcutaneous fat area; VFA, visceral fat area; SBP, systolic blood pressure; DBP, diastolic blood pressure; WC, waist circumference; HOMA, homeostasis model assessment; STU-1, Stumvoll first-phase insulin secretion index; STU-2, Stumvoll second-phase insulin secretion index; log, log-transformation; ACME, average causal mediation effect; ADE, average direct effect.

WGCNA was used to infer the co-expressed metabolite modules of closely interacting metabolites for clinical indices. The correlation between metabolite modules and clinical indices can help explain the clinical relevance of certain metabolite modules. At the same time, the significance of the correlation (*p* < 0.05) indicates the key role of metabolites in these modules (detailed module information is shown in Table S4). As shown in Figure 5A, the turquoise module is related to apolipoprotein A (APOA)/apolipoprotein B (APOB), TC, HDL and APOA in females, and this was also successfully validated in males (Figure 5B). These clinical indices are related to the lipid metabolism status of the body.

Although the role of the identified metabolite biomarkers in bone metabolism is still unclear, those metabolites that were included in the RF model for osteopenia had significant associations with a few relevant clinical indices. We found that several phosphatidylcholines are associated with clinical indices such as serum lipids, calcium, and pancreatic function in postmenopausal women (Figure 5C). Similarly, in males, lysophosphatidylcholine acyl C16:0, phosphatidylcholine diacyl C34:1, sphingomyeline C26:1, and some amino acids (including arginine, glutamine, ornithine, valine, and spermine) showed tight correlations with BMI, body fat mass percent, alkaline phosphatase levels, serum insulin, and serum lipids (Figure 5D).

Mediation analysis was further performed for the metabolites with the highest importance to investigate the mediating pathway from clinical indices to osteopenia or osteoporosis via metabolites. In females, the indirect effects of TFFM-log on osteopenia through three metabolites were significant, with an ACME of −0.01546 (95%CI: −0.21329, −0.00000) for phosphatidylcholine diacyl C38:5, −0.01558 (95%CI: −0.20869, −0.00000) for phosphatidylcholine diacyl C40:5, and −0.01702 (95%CI: −0.25712, −0.00000) for phosphatidylcholine diacyl C36:1, accounting for 47.9%, 47.6%, and 48.8% of the total relationship, respectively (Figure 5E, Table S5). Mediation analysis for osteoporosis in females and for osteopenia or osteoporosis in males found no significant mediating effect. Mediation analysis for other clinical indices also found no significant mediating effect.

## 4. Discussion

In this study, the serum metabolites were compared between three BMD groups, and visual separations of the metabolic profiles were observed according to the PLSDA in both men and women. Using iENA, several metabolites were found to have significant abundance and association changes in osteopenia participants, indicating that osteopenia is a critical stage in the development of osteoporosis. Thus, the metabolites showing differential levels between the normal and osteopenia groups were selected to build a machine learning model for the diagnosis of pre-osteoporosis. We found that the ability of key metabolites to discriminate osteopenia could be improved significantly in both men and postmenopausal women. Furthermore, we evaluated the association of metabolites with numerous clinical indices, with the result that several metabolites were found to be significantly associated with anthropometric and biochemical indices.

Osteopenia is a stage in the middle condition of fracture risk. The above analysis demonstrated that there are differences in the metabolic profiles between different BMD groups. Bone metabolism is a dynamic process related to the alteration of specific circulating metabolites. BMD changes are the consequence of bone metabolism. Several previous studies [38,39,40] have related serum or urine metabolomic profiles to BMD using a metabolomic approach, revealing that metabolites such as serum amino acids, serum lipids, and some urine metabolites (taurine, β-alanine, and 5-hydroxycaproic acid) are associated with BMD. Our results emphasize that osteopenia plays a critical role in the development of osteoporosis, with a particular involvement of phosphatidylcholine and amino acid metabolism in this process. Phosphatidylcholine metabolism is essential for the normal growth and mineralization of osteoblasts [41,42]; phosphatidylcholine concentrations of polyunsaturated fatty acids are also associated with hip BMD according to a 17-year follow-up study among older adults [43]. Spermine has been reported to be needed for bone development, and associated with lipid metabolism [44,45]. Motivated by this observation, we propose a novel and important hypothesis that osteopenia is a vital stage before osteoporosis; phosphatidylcholine and amino acid metabolism alterations in this stage might be promotional in bone loss. Correcting these metabolic disorders in this stage might be beneficial in preventing osteoporosis.

BMD assessment by DXA is the gold standard for diagnosing osteopenia and osteoporosis. However, novel serum or urine markers of bone status, which are predictive for bone loss and osteoporotic fracture, are arising. Although BTMs, including osteocalcin, PINP, and β-CTX, are useful in assessing the bone turnover status in osteoporosis diagnosis and the subsequent therapy process, they may not be the best markers for early warning in patients with osteopenia. Our results are also consistent with previous studies [24,46] and support the hypothesis that certain metabolites are potential biomarkers that could be used for the diagnosis and prediction of osteoporosis.

WGCNA revealed that metabolites were related to serum lipids. According to recent cross-sectional or cohort studies, serum lipid profiles have been found to be associated with BMD [26,35,36]. Bone also plays a significant role in lipid metabolism by regulating the clearance of circulating lipoproteins and non-esterified fatty acids [37]. Although the exact mechanism is not certified, our data also showed that there was a significant association between serum metabolism biomarkers and lipid profiles. Therefore, the metabolites in the turquoise module may be linked to BMD via lipid metabolism. The identified metabolite biomarkers in the RF presented similar clinical effects, and mediation analysis suggested that they contributed to mediating the association between TFFM and osteopenia. Our previous studies have found that TFFM was significantly associated with BMD, and mediation analysis based on metabolites possibly indicated the mechanism by which body composition influences osteoporosis. Therefore, taken together, these results indicate that serum metabolites are tightly associated with a person’s biochemical status. This finding may offer new insights into the role of metabolites in body metabolism and bone loss.

The metabolic predictors we identified are mainly phosphatidylcholine and some amino acids. The RF prediction model included five diacyl phosphatidylcholines and four acyl-alkyl phosphatidylcholines in females and one lysoPhosphatidylcholine and one diacyl phosphatidylcholine in males. Furthermore, phosphatidylcholine diacyl C34:1 was identified in the RF-predicted markers in both females and males, and it was also discovered in the iENA-related signal markers in females. A 17-year follow-up study among older adult women and men in the Framingham Osteoporosis Study found that plasma phosphatidylcholine essential fatty acids were associated with the femoral neck BMD and hip fracture risk [43]. Phosphatidylcholine is one of the most abundant phospholipids in biological membranes which mediates the transportation of fatty acids; it may play a role in the β-oxidation of the mitochondrial matrix, and it is beneficial for osteoblast lipid metabolism [47]. Previous studies have also found that serum phosphatidylcholine is essential for the function of osteoblasts [41,42]. In vitro studies indicated that glycerophospholipids promoted osteoclast differentiation, which was also correlated with a reduction in osteoblast differentiation and mineralization [48]. According to our WGCNA results, phosphatidylcholine diacyl C34:1 was also correlated with lipid-related indices. Although there is no direct explanation supporting the relationship between phosphatidylcholine diacyl C34:1 and bone metabolism, our findings offer the early alteration of phosphatidylcholine as a meaningful clue for a warning signal in osteoporosis, and we suspect that this might be via lipid metabolism. In addition, metabolites in the male model included four amino acids. All of them were negatively associated with osteopenia, which is consistent with previous research finding that several amino acids are beneficial for bone health by promoting the growth of bone and muscle cells, enhancing collagen formation, or affecting calcium homeostasis [49,50,51].

Gender differences have attracted much attention in bone metabolism. After menopause, women experience a sharp decline in estrogen levels, which leads to increased bone resorption and reduced bone formation, thereby increasing the risk of bone loss [52]. However, the decline in androgen levels in men is relatively slow, and its impact on bone is relatively small [53].

Our study still has some limitations, requiring further improvement in the future. First, as a cross-sectional population study, we only tested the serum metabolites and BMD values at one point, and the fracture rate was not included. Second, associations of these selected markers with bone loss need further replication in more studies, and the exact mechanism in bone metabolism needs to be certified in a basic study. Third, in this targeted metabolomic study, some metabolites may have been missed.

## 5. Conclusions

In conclusion, our study provides more evidence that osteopenia is a critical stage before osteoporosis and that metabolomic biomarkers can be used as predictive markers to detect osteopenia. Metabolomic biomarkers improve the ability to discriminate osteopenia, which provides new prevention guidance for osteoporosis.

## Data Availability

Data are available from the corresponding author on reasonable request.

## Supplementary Materials

The following supporting information can be downloaded at: https://www.mdpi.com/article/10.3390/metabo15010066/s1, Table S1. Metabolites showing trends in their levels in different groups; Table S2. Metabolite signatures detected by iENA in females and males separately; Table S3. Differential metabolites between the control group and osteopenia group in females and males separately; Table S4. Metabolite module extracted by WGCNA using female and male data separately; Table S5. Mediation analysis of identified metabolites between TFFM and osteopenia in females.
